# Simple Method for the Objective Activity Type Assessment with Preschoolers, Children and Adolescents

**DOI:** 10.3390/children7070072

**Published:** 2020-07-02

**Authors:** Jan Christian Brønd, Anders Grøntved, Lars Bo Andersen, Daniel Arvidsson, Line Grønholt Olesen

**Affiliations:** 1 Center for Research in Childhood Health/Unit for Exercise Epidemiology, Department of Sports Science and Clinical Biomechanics, University of Southern Denmark, 5230 Odense M, Denmark; agroentved@health.sdu.dk (A.G.); lgolesen@health.sdu.dk (L.G.O.); 2 Faculty of Teacher Education and Sport, Sogn og Fjordane University College, 6856 Sogndal, Norway; lars.bo.andersen@hvl.no; 3 Department of Food and Nutrition and Sport Science, Center for Health and Performance, University of Gothenburg, 405 30 Gothenburg, Sweden; daniel.arvidsson@gu.se

**Keywords:** classification, activity type, acceleration, thigh

## Abstract

**Background:** The objective and accurate assessment of children’s sedentary and physical behavior is important for investigating their relation to health. The purpose of this study is to validate a simple and robust method for the identification of sitting, standing, walking, running and biking performed by preschool children, children and adolescents in the age from 3 to 16 years from a single thigh-worn accelerometer. **Method:** A total of 96 children were included in the study and all subjects followed a structured activity protocol performed in the subject’s normal kindergarten or school environment. Thigh acceleration was measured using the Axivity AX3 (Axivity, Newcastle, UK) device. Method development and accuracy was evaluated by equally dividing the subjects into a development and test group. **Results:** The sensitivity and specificity for identifying sitting and standing was above 99.3% and for walking and running above 82.6% for all age groups. The sensitivity and specificity for identifying biking was above 85.8% for children and adolescents and above 64.8% for the preschool group using running bikes. **Conclusion:** The accurate assessment of sitting, standing, walking, running and biking from thigh acceleration and with children in the age range of 3 to 16 is valid, although not with preschool children using running bikes.

## 1. Introduction

Children and adolescents spend, on average, 6–8 h of their time awake on sedentary behavior (SB) [1], and engaging in excessive amounts of SB may be adversely associated with physical and mental health [2]. Different determinants of SB have been investigated and active transportation seems to be one modifiable component that may reduce sedentary time in the transition from childhood into adolescence [3]. The accurate and objective assessment of SB and biking activities with children and adolescents would be valuable to further investigate the determinants of SB in this population. 

Assessing physical activity and SB in children and adolescents is challenging [4], and objective assessment using activity monitors worn at the wrist or hip has been shown to be an attractive method when compared to both direct observation and self-report methods [5]. The validity of using the hip [6] or wrist [7,8], respectively, for the assessment of SB has been investigated, and significant differences have been reported [9]. Using the wrist has been shown to provide an excellent wear comfort, which increases subject adherence to the measurement protocol [10], but thigh-worn accelerometers have also been shown to provide high compliance rates with tape-mounted devices [11,12]. The ActivPAL (PAL Technologies Ltd., Glasgow, UK) activity monitors are taped to the thigh and have been shown to provide accurate estimates of SB based on the assessment of postural allocation with children and adolescents [13,14,15]. The underlying algorithms for identifying SB with the ActivPAL device are not available to the researcher, and the lack of transparency makes it difficult for researchers to fully understand the strength and limitations of the selected methodology. Furthermore, the economic burden of using the ActivPAL device might make it more applicable with studies including a small number of subjects. These limitations with the ActivPAL device have previously been addressed by Skotte et al. [16] proposing an open source method for the identification of the following body positions and activities: Sitting, standing, walking, running and biking with adults using a single ActiGraph GT3X (ActiGraph LLC, Pensacola, FL, USA) accelerometer worn on the thigh [16]. However, the size and bulky design of the ActiGraph GT3X device does not make the proposed method by Skotte et al. [16] applicable for use with smaller children. In the study by Stewart et al. [17], a smaller open source Axivity AX3 (Axivity, Newcastle, UK) device was used for the identification of children’s activity types using accelerometers worn at the thigh and trunk in addition to the use of a more advanced machine learning method in the data processing [17]. However, besides the use of an advanced method, the study only provided data for children 7–15 years of age and did not investigate the identification of biking. Moreover, the underlying implementation of this method, and also the method proposed by Skotte et al. [16], have never been made public available, which is a major limitation. Currently, there are no simple and public methods available for the assessment of PA behavior—specifically SB and biking—with preschoolers, children and adolescents using a single thigh-worn activity monitor.

The aim of this study is to investigate the accuracy and validity of a simple method for the identification of six common activity types with preschoolers, children and adolescents using a single accelerometer worn on the thigh, and to make this method publicly available as open source.

## 2. Methods

### 2.1. Participants

A total of 96 Danish children were included in the study. Twenty-nine preschool children (age three to six) were recruited from a local kindergarten in the Aarhus Municipality in 2018, whereas 36 children (third and fourth grade) and 31 adolescents (eight and nine grade) were recruited from a local school in the Odense Municipality. Written information about the study and an invitation to join the oral information meeting arranged for the children at the preschool were communicated out using the preschool–parent communication internet platform and handouts. During the study period, it was secured that the preschool children having the parents´ written consent also gave their assent. This was secured being aware of the children´s reactions, and whether they seemed to be comfortable or not with the test situation.

The children and adolescents were invited by email through the school office and word of mouth. Informed consent was provided by their parents The Ethics Committee of the Region of Southern Denmark approved this additional preschool study, as a part of the Motor skills in PreSchool study (S-2015-0178) as well as by the Danish Data protection Agency (10.450). The Ethics Committee of the Region of Southern Denmark also approved the children and adolescent study (S-20140068).

### 2.2. Procedure

The protocols for the preschool children and the school children are not identical but in both studies all children performed the activities in the same order as presented in Table 1. 

All subjects performed the protocol using the same order (1–11) and the basketball activity was not performed by the preschool children and the swing activity was not performed by the children and adolescents group. The intensity classification is based on the measurement of energy expenditure of similar activities in young children [18]. The total duration of each activity was 1.5–5 min. A shorter duration (1.5–3 min) was used with the preschool children than with children and adolescents (5 min). This was to ensure the preschool children could sustain the activity for the full duration. The intensity of each of the movement activities was self-selected, but subjects were also encouraged to adapt to an intensity they could complete the suggested distance (preschool study) or full duration of each activity (school study). All sedentary activities were performed indoors and all other activities outdoors at or around the preschool or school area, respectively. An activity log was used to record activity start times (timestamps using seconds accuracy) during protocol execution and subsequently used to extract the activity specific information with the analysis. The log time was determined from the researcher’s smart phone which was synchronized with the computer that was used to initialize the accelerometers. The activity start times were manually validated after data download using visual inspection of the raw acceleration. If the start time was more than 5 s from the onset of the activity, it was adjusted to the exact start of the activity. The activities basketball, playground and swing are included to assess the performance of the method with activities that are a complex mixture of fast transitions but also movement behaviors that are difficult to define. 

#### 2.2.1. Preschool Children 

Most children were tested one at a time, but some were tested two at the time. No height, weight or anthropometric values were measured, as this was not specified in the protocol accepted by the ethics committee. All children willing to participate was included in the study. During the assessment process, attention was made to the children’s visual appearance (height and weight) to assess if this was a reasonable homogeneous preschool group. The indoor activities were carried out in different locations at the preschool throughout the test period. Thus, the children were sitting and standing at different chairs and tables, but arrangements were made to secure somewhat appropriate chair and table sizes. To avoid too many disturbances, the outdoor activities were carried out on a nearby flat, less busy walking and bicycle path with hard surface running through a small recreative area. The activities were carried out on a circular path with a distance of 75 m/246 feet. Each child was joined by the researcher during the activities and encouraged to perform 1–4 laps at a time depending on activity type, age and selected speed. One child performed all the outdoor activities at the preschool playground. During the indoor sitting and standing activities, the child could choose his/her own sitting position. The time was stopped if the child changed body position—e.g., from sitting to standing position—but not if the child adjusted sitting position. In the standing position, the time was stopped if the child stepped outside the predefined area (a hula hoop) or picked up toys dropped on the floor. If the child stopped or changed activity during the outdoor activities the time was stopped and continued after a small break. The position of the thigh belt was checked before and after performing an activity, since the monitors were not mounted with tape. In total, the whole procedure for each child took, on average, one hour (range 48–85 min), and the children completed 9–10 activities with each activity lasting around 1.5–3 min. 

#### 2.2.2. Children and Adolescent 

The measurements presented in Table 1 were performed right after school. The participants were introduced to the activity protocol right after arrival to an empty classroom following the measurement of their body weight on a calibrated digital scale and body height using a stadiometer. Anthropometric measurements of arm, leg and waist circumference were measured with a soft tape measure. Arm length was measured from the acromion to the tip of the middle finger, and leg length as the distance between the iliac crest to the floor while standing in an upright position with legs together. All equipment was attached to the body and the measurement started after an adaption period of 10 min. The walking, fast walking and running activities were performed consecutively in order of intensity without breaks. A 2–5-min natural break was used between all other activities. The duration of each activity was 5 min and the first 60 s of data was not used in the analysis.

### 2.3. Instrumentation

The Axivity AX3 (Axivity, Newcastle UK) is a small (23 × 32.5 × 7.6 mm) MEMS based triaxial accelerometer with 512Mb of onboard memory weighing only 11 g. The device is IPx8 certified providing water resistance at 1.5 m for 1 h. The Axivity AX3 (Axivity, Newcastle UK) provides a configurable sampling frequency (12.5–3200Hz), measurement range (±2, ±4, ±8, ±16 g) and 12-bit resolution (13-bit using the ± 16 g measurement range). The battery capacity of the Axivity AX3 provides measurement of 14 days at a 100 Hz sampling frequency. The OMGui (current version 1.0.0.30) used with instrument initialization and data download is available online [19]. The subjects included wore the accelerometer on the right front thigh at the same location as used in the original study by Skotte et al. [16]. The orientation of the device for the preschool children was with the *y* axis pointing towards the knee and *x* axis lateral (label side of the device visible and USB connection lateral); whereas for the children and adolescents, the orientation was with the *x* axis pointing towards the knee and *y* axis lateral (label side of the device visible and USB connection pointing towards knee). The monitors were attached using belts made of OEKO-TEX-certified materials with the preschool children and tape mounted on the skin using a hospital dressing commonly used with wound treatment for children and adolescents [11]. The alternative device orientation used with the preschool children was needed to reduce belt width, and thus improve wear comfort. Device orientation is specified with the classification method implemented in Matlab, which will automatically account for the orientation. All instruments were initialized to measure acceleration using 100 Hz sampling frequency and ±8 g measurement range. The acceleration was resampled after data download to the same 30 Hz sampling frequency used in the study by Skotte et al. [16]. The downsample function available in Matlab was used to resample the data.

### 2.4. Classification Method

**Figure 1:**
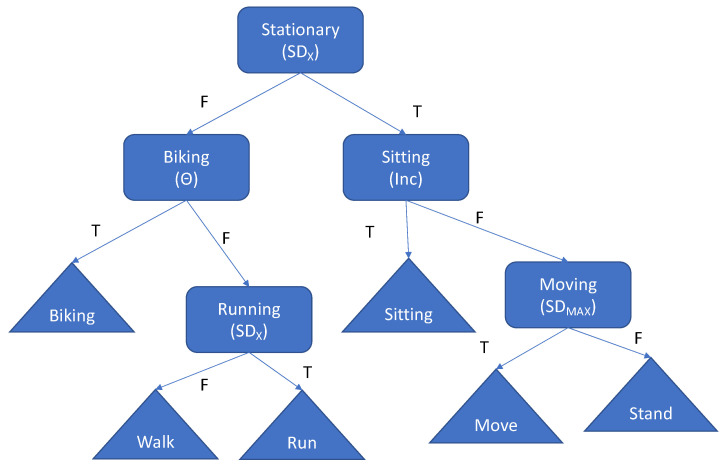
Decision tree implemented for identifying sitting, standing, walking, running, biking and moving (T = true, F = false). SD_X_ is the standard deviation of the acceleration in the x direction, Θ is the backward/forward angle, Inc is the inclination angle and SD_MAX_ is the maximum standard deviation of the acceleration across all axes.

The identification of sitting, standing, walking, running and biking using the acceleration measured with a single thigh-worn accelerometer is implemented as a simple decision tree. This is the same method used in the study by Skotte et al. [16], although not including stair walking. It is not the purpose of this study to evaluate the accuracy of using the adult thresholds with children but to assess new thresholds and the corresponding accuracy. A movement category is introduced in the method to categorize activities performed in an upright posture, which include minor movements. This could be preschool children’s playing in the sandbox making sand-cake at the outdoor child kitchen, or washing dishes or folding laundry by either adolescents or children. The outline of the decision tree is presented in Figure 1. 

A total of five conditions are required to identify the six activity types. The signal features used with the five conditions are: The standard deviation of the acceleration in the *x*-axis (SDx—along the thigh direction), inclination angle (Inc), maximum standard deviation across all axes (SD_MAX_) and backward/forward angle (Θ). All signal features are generated for each 2-s window using a 50% overlap across the complete recording, which provides a temporal classification resolution of 1 s. The first condition in the decision tree discriminates into either a stationary or dynamical movement branch using the SDx signal feature. The first condition in the stationary branch discriminates between the sitting and standing postural allocation using the inclination angle, which subsequently discriminates into standing still and moving using the SD_MAX_ feature. The first condition in the dynamical movement branch uses the angle feature to discriminate between the biking and locomotion, and subsequently the locomotion into walking or running using the SDx. The median filtering used in the original study by Skotte et al. [16] to eliminate sporadic misclassification is also implemented in this proposed method. The identification of lying was implemented as proposed in the study by Skotte et al. [16] combining the inclination angle of the back (>65 degrees) in combination with the identification of sitting with the thigh. The identification accuracy of lying was not evaluated. Multiple assignments of activity types caused by the median filtering are not permitted and reduced to the activity that seem most obvious. Thus, if lying or sitting is identified, then any other activity is removed. If biking and other activities are identified, then biking is selected. If either walking or running in combination with either stand or move is identified, then either walking or running is selected. 

### 2.5. Statistical Considerations

The conditional thresholds of the decision tree and accuracy of the method were investigated by dividing all subjects into a development or validation group. One half of each age group was randomly selected for the development group, with the rest of the subjects assigned to the validation group. The activity log recorded during the measurement protocol was used to extract the activity specific signal features. 

The threshold used to discriminate between dynamical movement and stationary activities using SD_X_ was evaluated using the pooled distribution of all sitting and standing activities with respect to all activities requiring dynamical movements of the whole body (walking, running basketball, playground). All pooled distributions were plotted with the ggplot2 R package and specifically the geom_density function. The default setting of this function was used. The threshold for identifying biking from locomotion using the backward/forward angle (Θ) was determined by evaluating the pooled distribution of biking with respect to all locomotion activities. The threshold for the identification of running from walking using SD_X_ was evaluated by the pooled distribution of running in relation to both the normal and brisk walking activities. The threshold for identifying sitting from standing activities using the Inc angle was determined from the pooled distribution of all sitting activities with respect to all standing activities. The threshold for identifying standing still from moving around (shuffling) using SD_MAX_ was determined by evaluating the pooled distribution of all standing activities with respect to the normal walking activity. The accuracy of identifying the activities sitting, standing, walking, biking, and running was assessed by evaluating the sensitivity and specificity determined from the agreement between the expected activity type and estimated activity (tabulated data) using the second by second level data. For evaluating method performance with basketball, playground, and swing, the relative amount of individual activities is presented. All accelerometry data processing and activity type identification was implemented in Matlab (Mathworks Inc. Version R2019a 9.6.0) and is publicly available for download on Github (https://github.com/jbrond/SkotteChild). All statistical analyses were performed using R (Version 3.5.1).

## 3. Results

### 3.1. Descriptive Statistics

The age of the preschool children ranges from 3 to 6 years, 9 to 12 years in the children group, and 13 to 16 in the adolescent group. The height and weight of the preschool children were not measured, but none of the included subjects seemed to have a stature or weight that was substantially different from the general norm of Danish preschool children. The mean (SD) weight of the children and adolescents was 38.7 (7.0) kg and 59.2 (9.6) kg, and height was 145 (7.3) cm and 170.8 (11.1) cm, respectively. A pairwise T-test showed no significant difference in the age, weight, height or any of the anthropometric values between the development and validation group.

### 3.2. Algorithm Development

**Figure 2:**
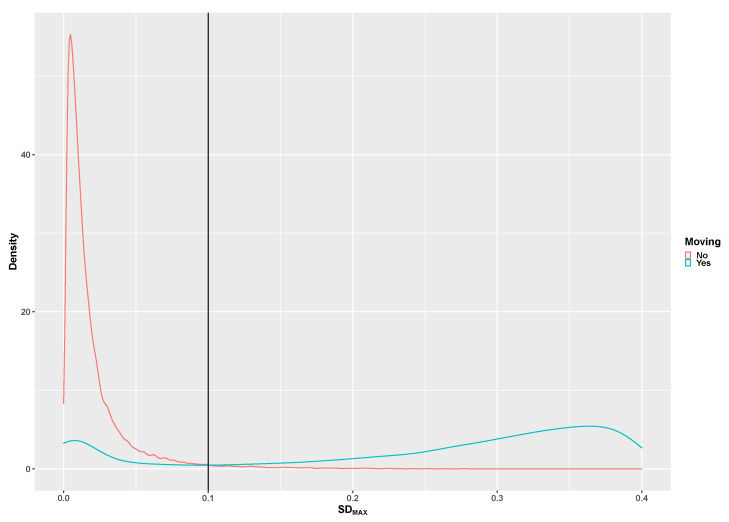
The pooled distribution of the SD_MAX_ signal for moving (green curve) and non-moving (red curve) activities. The vertical line represents the original adult 0.1 g SD_MAX_ threshold [16].

**Figure 3:**
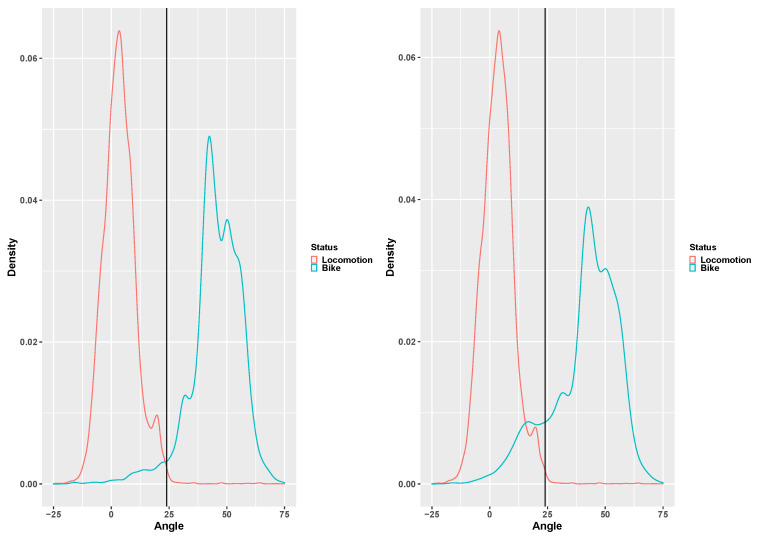
The pooled distribution of the angle signal feature for locomotion (red curve) and biking (green curve) only including the children and adolescents group in the left plot and all groups included in the right plot. The vertical line represents the original adult 24-degree threshold [16].

**Figure 4:**
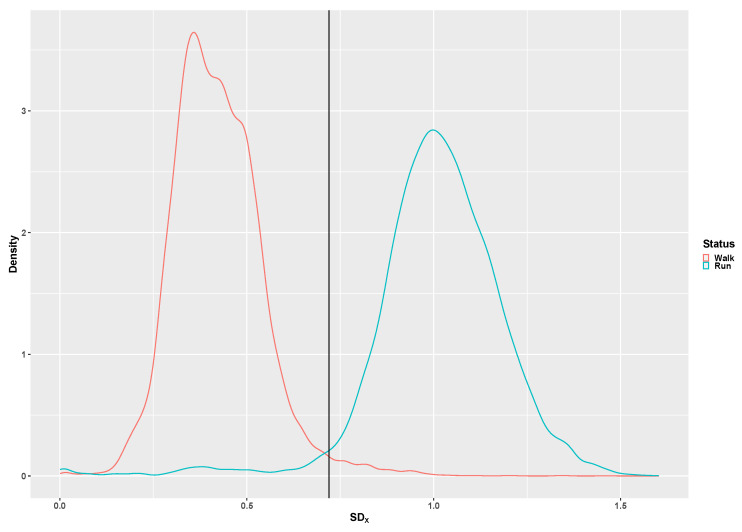
The pooled distribution of the SD_X_ signal feature for walking (red curve) and running (green curve). The vertical line represents the original adult 0.72 g SD_X_ threshold [16].

**Figure 5:**
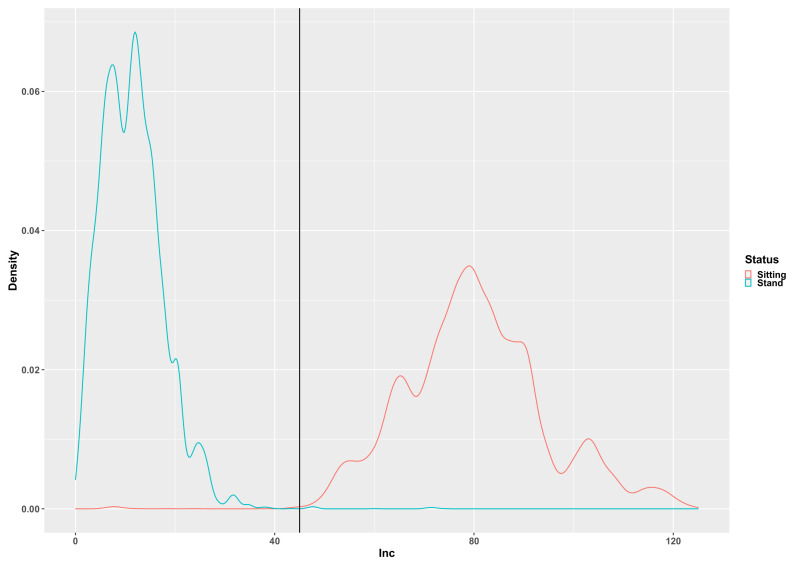
The pooled distribution of the Inc (inclination angle) signal feature for sitting (red curve) and standing (green curve) activities. The vertical line represents the original adult 45.0 degrees threshold [16].

**Figure 6:**
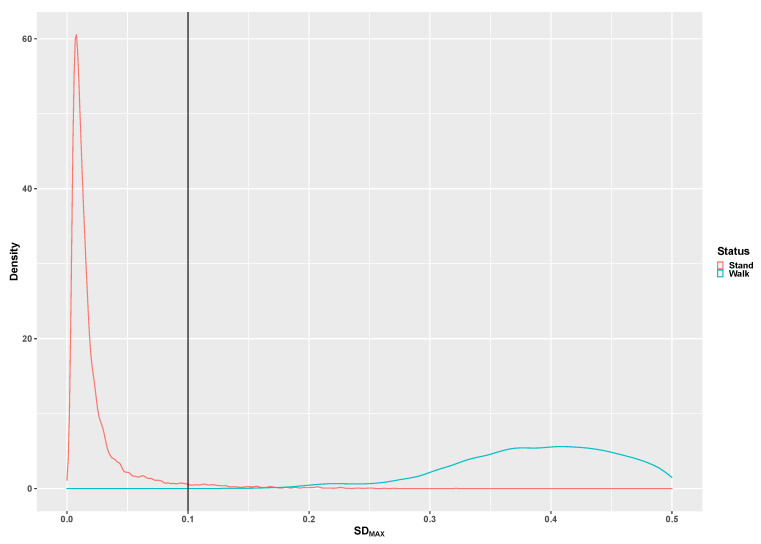
The pooled distribution of the SD_MAX_ signal feature for standing (red curve) and walking (green curve) activities. The vertical line represents the original adult 0.1 g SD_MAX_ threshold [16].

Figure 2, Figure 3, Figure 4, Figure 5 and Figure 6 illustrate the calculated signal features to discriminate between the tested activities based on data from the developmental group. The original adult thresholds determined by Skotte et al. [16] are presented in all figures (black vertical line) for comparison. The optimal threshold to be used with the preschool children, children and adolescents was made by the visual inspection of the distribution in combination with the threshold known from the adults. Different analytical methods were explored (Receiver Operating Characteristics, Naïve Bayesian classification). However, the thresholds determined from these methods were very different from those of the adults, which indicated that an analytical method is not optimal. The SD_x_ distribution for the stationary and dynamical movements activities is presented in Figure 2. The 0.1G SD_x_ threshold determined with adults is similarly applicable to preschool children, children and adolescents. 

The angle distribution for biking and locomotion activities is presented in Figure 3, with only children and adolescents in left plot and all the age groups included in the right plot. From the right plot it is clear that the proportion of angle below 25 degrees is substantially increased by adding the preschool children. However, the optimal angle threshold for discriminating biking from locomotive activities is 22.5 degrees, which is slightly lower than the 24 degrees used with adults. Setting the detection of biking at this threshold seems to suggest an increased misclassification with biking in the preschool group as compared to the children and adolescents’ groups. This is most likely caused by the use of running bikes with the preschool children and is further addressed in the discussion. 

The SD_x_ distribution for walking and running is presented in Figure 4. The SD_x_ threshold for discriminating walking from running is 0.65G, which is lower than the 0.72G determined with adults. 

The inclination angle distribution for the sitting and standing activities is presented in Figure 5. The optimal inclination angle discriminating sitting from standing is 47.5 degrees, which is slightly higher than the 45 degrees used with adults. 

The SD_MAX_ distribution for standing still and locomotion activities is presented in Figure 6. The SD_MAX_ threshold for discriminating standing still from moving/shuffling is 0.13G, which is slightly higher than the 0.1 found with adults. 

### 3.3. Algorithm Validation

Applying the identified thresholds in the decision tree with the validation group resulted in a sensitivity above 64.8% and specificity above 95% for all activities. The individual activity related sensitivity and specificity for each age group are presented in Table 2. 

The sensitivity and specificity are above 99% for sitting and standing activities across all age groups, and the sensitivity and specificity for the remaining activities biking, walking and running is above 85.8% or close to 100% for children and adolescents. The sensitivity for biking is only 64.8% with the pre-school children, which is lower than with children and adolescents. The basketball activity performed by the children and adolescents was identified as walking (62%) with only 28.7% of time identified as running. The playground activity performed by nine school children was mainly identified as walking (83.5%), with only 8.5% identified as running. A minor part of the basket and playground activities (<1%) were identified as moving and standing. The playground activities performed by the preschool children were identified as walking (40.9%), moving (28.0%), biking (12.8%), standing, (9.8%), running (8.2%), and sitting (0.3%). The swing activities were identified as biking (50.0%), sitting (38.7%), standing (7.9%), and moving (3.4%).

## 4. Discussion

In this study, we evaluated a method for identifying sitting, standing, moving, walking, biking and running using a single Axivity AX3 (Axivity, Newcastle UK) accelerometer worn on the thigh with preschoolers, children and adolescents. The results demonstrate that the proposed method provides an excellent sensitivity and specificity with all the proposed activities, with the only exception for biking on running bikes with pre-school children. The accuracy found in this study is comparable to the original study conducted on adults using the same method for the identification [16]. 

Other studies have evaluated techniques to identify children’s time spent in different activity types from accelerometer data. Trost et al. [20] used logistic regression to identify seven activity types (lying, sitting, standing, walking, running, basketball, and dancing) in 52 children and adolescents using accelerometers worn either on the hip or wrist. The hip- and wrist-based models achieved 91.0% and 88.4% accuracy, respectively. In a study by Stewart et al. [17] a dual-accelerometry system was evaluated with both children and adults for the identification of six activity types. One device was worn at the thigh and another device worn on the lower back. A random forest algorithm and a total of 142 different signal features generated from the raw acceleration were used in the identification of activity type. The random forest algorithm is an ensemble learner and performs the identification of activity types from acceleration using multiple individual decision trees [21]. The method used in the present study only uses a single decision tree and just six different signal features. Despite the large difference in algorithm complexity and number of features, the sensitivity and specificity are above 98% for most activities in both studies. Thus, using a more complex algorithm or additional features does not seem to improve the identification accuracy per se, suggesting that wear location and optimal selection of signal features might be more important than the algorithm and number of signal features.

Including biking in the identification of activity type is primarily due to the known health benefits of this activity, but also due to the important assessment of transportation mode which is great importance in many countries. The lower sensitivity and specificity demonstrated for the identification of biking with preschool children is clearly caused by the use of running bikes rather than actual bikes with pedals. Running bikes do not have pedals as normal bikes do, and driving the running bike forward is carried out by either “running”, or double pushing both legs on the ground, or simply by having a break resting the legs on the footrests, although the children were encouraged not to do so. The accuracy for identifying the running bike as biking could be increased by decreasing the forward/backward angle threshold in the identification of biking from locomotion. Decreasing the angle threshold, however, would also potentially increase the misclassification of some running activities as biking, which will decrease the sensitivity of identifying locomotion in real data. The acceleration not identified as biking with the preschoolers using a running bike is primarily identified as running, but also as walking. Considering the actual movement with the running bike, it is more correct to the actual movement performed rather than as an indented biking activity. 

The activities included in the present study and the study by Stewart et al. [17] are performed in controlled environments, which simplifies the data processing and analysis. However, the frequent transitions between activities—which are an important element of children’s common movement behavior during free-living—are not included, suggesting that the sensitivity and specificity estimated by Stewart et al. [17] and in the present study might not accurately reflect the performance of the algorithms in a free-living environment. In an attempt to address this, we included a basketball activity with the children and adolescents, and a playground activity with the children and preschoolers. The basketball and playground activities included movements such as standing still, moving, walking, running and jumping, varying in both duration and organization. We did not assess the amount of time spent with the different activity categories with the basketball and playground activities, but the estimated duration of the individual activity types seems to reflect the overall nature to be expected of these activities. The method proposed by Stewart et al. [17]was evaluated in a free-living environment, and the results provide excellent sensitivity and specificity with most activities [22]. However, transitions were excluded from the analysis and the results also further indicate that the identification of dynamic standing and movement is challenged with children. Increasing the number of available features and the complexity of the algorithm will increase the risk for overfitting and thus misclassification of some activity types in a free-living environment. The complex and sporadic movement behaviors, especially of younger children, seem to suggest that a robust identification (balance between sensitivity and specificity) of common activity types with children is most optimally approached using a limited number of signal features. The children enrolled in the free-living evaluation by Steward et al. [17] were at the age of 10 years, and further studies seem to be required to evaluate the classification accuracy with even younger children in a free-living environment. Moreover, the implementation of the proposed method described by Stewart et al. [17] is not publicly available, which makes it difficult for other researchers to use, replicate and modify. 

The identification of stair walking was included in the original method proposed by Skotte et al. [16]. The identification of stair walking was implemented using an individual defined threshold, determined using the median value for the forward/backward angle (Θ) below 5 degrees, and adding 4.5 degrees (Θ_d_ = Θ_m_ + 4.5). The threshold for the identification of stair walking is therefore <9.5 degrees, and thus sensitive to the misclassification of walking and running as stair walking. Running and walking movements with children are commonly performed in a complex environment and, combined with children’s short legs, it seems to increase the risk for the children to generate backward/forward angles that resemble stair walking. The question of whether to include or exclude stair walking is a balance between the misclassification of stair walking as normal walking or the misclassification of walking and running as stair walking. Most children do perform stair walking, but considering the nature of movement in children, we might introduce a systematic bias by the misclassification of walking and running as stair walking rather than obtaining an accurate estimate of children’s stair walking. In some environments, stair walking might be an important element of children’s movement behavior, which requires the accurate quantification of stair walking. However, only including the forward/backward angle in the identification of stair walking seems inadequate for obtaining a robust identification with younger children. This could argue for using additional features, as with the study by Stewart et al. [17] for the identification of stair walking. However, as previously mentioned, increasing the number of signal features increases the risk of overfitting, and thus the misclassification of the actual activity performed. Currently, there is no single robust signal feature available for the accurate identification of stair walking in children, and further investigation of the biomechanical properties of children’s stair walking in relation to the acceleration measured at the thigh seems to be required. 

All signal features generated in the method proposed by Stewart et al. [17] are determined using a 5-s non-overlapping window, whereas only a 2-s 50% over-lapping window (providing second by second resolution) is used in the method proposed by Skotte et al. [16] and the present study. The need to use a longer time window with the method proposed by Stewart et al. [17] is most likely to provide sufficient resolution with the frequency-related signal features, in order to accurately detect the cyclic or non-cyclic nature of some activities. The dominant frequency is commonly included in many machine learning algorithms [17,23], and estimated using the Fast Fourier transformation (FFT). The resolution and minimal detectable frequency is coupled with the total number samples used to estimate the feature. The sporadic nature of children’s movement behavior in a free-living environment seems to suggest a general increased misclassification, with longer time windows as compared to shorter time windows. Window size or epoch length has previously been investigated extensively with intensity-derived measures from acceleration, and it is clear that the intermittent nature of children’s activity has to be analyzed with short-duration epochs [24,25]. Another interesting difference between the present study and the study conducted by Steward et al. [17] is the use of non-overlapping and overlapping windows. Non-overlapping windows determine the activity type for each window independently, whereas over-lapping windows consider a smoother transition between windows. Preschoolers and children often perform a transition between different activity types lasting less than 5 s, and most likely not in synchronization with the window by window classification [26]. This will cause the rate of misclassification to follow the number of activity transitions performed by the subject. However, using over-lapping windows might also be challenged with identifying the actual onset and offset between activities. The optimal selection of window size and overlapping windows seems to be an important aspect of the accurate classification of activity types in children, which seems to require further investigation in the future. 

The lying posture is commonly interpreted as an indirect measure of sleep and the identification of lying using accelerometry has been approached using both single and multiple devices worn on both wrist, thigh, and hip [17,21,27,28]. For many children and adolescents, it is not uncommon in the late afternoon or evening, during weekdays or generally during weekend days, to lie on the couch or in bed watching TV, using a tablet or their cell phone. This seems to suggest that with the indirect measure of sleep from the identification of lying, as validated with laboratory conducted experiments, there is a substantial risk of misclassifying sedentary behavior as sleep. The lying posture allocation associated with evening sedentary behavior is clearly not sleep and should not be included as such. The accurate identification of lying and sleep with 24-h free-living recordings is challenging and only including a laboratory lying activity, even though it is in different positions, does not provide sufficient information for distinguishing lying as sedentary behavior from time in bed and thus sleep. If accelerometry is to be used to provide an indirect assessment of time in bed and potentially sleep, it is of utmost importance to distinguish between these behaviors. The accurate identification of lying and time in bed/asleep has to be performed using measurements conducted with subjects during their free-living behavior similar to in previous studies [29,30,31,32,33], and thus including the important temporal information regarding circadian rhythm and essentially sleep behaviors. Currently, there is only one study investigating the assessment of sleep using free-living recordings and a thigh-worn device. However, this study relies on the proprietary ActivPAL device and the validity was assessed with adults [28]. Future studies should investigate the accurate identification of children’s lying from time in bed with thigh-worn accelerometers using free-living recordings, rather than only relying on laboratory data or standardized protocols.

### Strength and Limitations

A major strength of this study was that all activities were performed in the subject’s natural environment, as well as the inclusion of subjects across multiple age groups. Although the subjects followed a strict protocol during the field validation, we allowed some natural movement adjustment during most activities—for example, adjusting sitting position when sitting. The use of short and overlapping windows in the generation of the signal features is a strength of the method, and provides a resolution which is required with the sporadic nature of this population. The method implementation is made publicly available and open source.

A major limitation with the present study that a free-living validation is not included. Conducting a true free-living validation is challenging, and the sensitivity and specificity estimated in this study do not reflect the true sensitivity and specificity with the method using real recordings. In the discussion, we addressed the possible limitations of the current method in comparison to more advanced methods and, considering the complexity of human behavior and movement, it seems to suggest that selecting a simpler and more robust method might perform better with real recordings. It is a minor limitation to use running bikes to perform biking for preschool children. However, the number of preschool children capable of using a real bike with pedals is likely to be very small, suggesting that this activity is not common with real recordings. It is a limitation that the method is implemented in the commercial and costly software Matlab^®^. However, GNU Octave (https://www.gnu.org/software/octave/) is a free and open-source alternative to Matlab. We implemented the described method using standard functions which are also available with Octave. Moreover, using a standard function also provides an easy replication of the method with common statistical software such as R or Python, which are freely available.

## 5. Conclusions

The identification of six common activity types with preschoolers, children and adolescents with a single accelerometer worn at the thigh has been presented and evaluated. The cross validation demonstrated an excellent sensitivity and specificity, suggesting that the proposed method is valid for determining the time spent in different activity types in preschoolers, children and adolescents in a free-living environment. 

## Figures and Tables

**Table 1:** Activities performed during the structured protocol at the school and preschool study.

Order	Intensity Category	Activity	Description of Activity School Study	Description of Activity Preschool Study
1	Sedentary	Sitting	Sitting on a chair close to a table with arms in the lap	* Sitting on the buttocks on the floor playing with Lego or Geomac toys.
2	Sedentary	Sitting playing	Playing the Fruit Ninja game on an iPad	Sitting on a chair close to a table playing with Lego or Geomac toys at the table.
3	Light	Standing playing	Playing a game on the iPad while standing	* Standing close to a table playing with Lego or Geomac toy at the table.
4	Light	Slow walking	Slow walking speed	Walks at the child’s preferred walking speed together with the instructor.
5	Moderate	Brisk walking	Brisk walking speed	Walks fast trying to catch up with the instructor without running.
6	Vigorous	Running	Running at the subjects own preferred running speed	Runs at the child’s preferred running speed together with the instructor.
7	Very vigorous	Basketball	One-to-one competitive basketball game play	Not performed
8	Very vigorous	Playground	** Running and walking around the school playground in a follow my leader activity	Walking, crawling, jumping and running through a predefined obstacle course
9	Moderate/vigorous	Biking	Commuting cycling on subjects’ own bike	Cycling on an adjustable child running bike at the child´s preferred speed.
10	Sedentary	Sitting	Sitting close to a table with arms in the lap	
11	Light	Swing	Not performed	***Sitting on a swing in self-selected effort

* The child is placed in a hula hoop and told not to step out of it while playing ** This activity was only performed by nine subjects from the children’s group *** The activity was only performed by nineteen subjects from the preschool children´s group.

**Table 2:** Activity type identification accuracy for sitting, standing, biking, walking and running for the three age groups—preschool, children and adolescents—using the validation data.

	Pre-School		Children		Adolescents	
	Sensitivity	Specificity	Sensitivity	Specificity	Sensitivity	Specificity
Sitting	100.0	100.0	99.8	99.7	100.0	99.3
Standing	100.0	99.8	100.0	99.8	100.0	100.0
Biking	64.8	100.0	85.8	100.0	94.9	100.0
Walking	82.6	98.1	93.3	100.0	100.0	99.9
Running	92.4	95.0	99.9	97.3	99.6	99.9
